# Genomic and structural insights into the in vivo development of cefiderocol resistance in Klebsiella pneumoniae

**DOI:** 10.1007/s00253-026-13967-y

**Published:** 2026-07-24

**Authors:** Alexander Tristancho-Baró, Ana Isabel López-Calleja, Ana Milagro-Beamonte, Blanca Fortuño, Juan Manuel García-Lechuz, Rosa Martínez, Ruth Caballero, Miriam Latorre-Millán, Laura Clusa, Lilla Buzgó, Carmen Torres, Antonio Rezusta

**Affiliations:** 1 Biomedical and Biotechnological Sciences Program, University of La Rioja, 26006 Logroño, Spain; 2 Difficult-to-Diagnose and Treat Infections Research Group, Institute for Health Research Aragon, Miguel Servet University Hospital, 50009 Saragossa, Spain; 3 Clinical Microbiology Laboratory, Miguel Servet University Hospital, 50009 Saragossa, Spain; 4 Department of Microbiology, Faculty of Medicine, University of Zaragoza, 50009 Saragossa, Spain; 5 Infectious Diseases Department, Miguel Servet University Hospital, 50009 Saragossa, Spain; 6 Department of Infection Control and Hospital Epidemiology, Faculty of Health Sciences, One Health Institute, University of Debrecen, 4032 Debrecen, Hungary; 7 Area of Biochemistry and Molecular Biology, One Health-UR Research Group, University of La Rioja, 26006 Logroño, Spain

**Keywords:** Cefiderocol resistance, Whole-genome sequencing, Comparative genomics, Carbapenem-resistant Enterobacteriaceae, Structural modelling

## Abstract

**Abstract:**

Cefiderocol is a newly developed siderophore-conjugated cephalosporin specifically designed to overcome multidrug-resistant (MDR) Gram-negative bacilli. Nevertheless, reduced susceptibility and resistance to cefiderocol has increasingly been reported, especially derived from alterations in iron transport systems and plasmid-mediated determinants and the expression of structural variants of β-lactamases. Moreover, cefiderocol resistance derived from target alteration remains insufficiently defined. This work aimed to characterize the genomic and structural basis of cefiderocol resistance emerging in vivo during therapy in *Klebsiella pneumoniae*. Comparative genomics revealed conserved plasmid architecture and no acquisition of novel β-lactamases or iron-uptake determinants. Eighty-two chromosomal variants were identified, among which a missense substitution in ftsI (FtsI G306V), located adjacent to the catalytic serine (S307) of PBP3, emerged as a plausible resistance determinant. Structural modelling suggests that the G306V substitution introduces a significant side-chain volumetric increase, resulting in locally increased steric hindrance within the active-site, possibly impairing cefiderocol interaction with its target. The absence of canonical iron-transport alterations further distinguishes this case from the predominant mechanism described in global molecular epidemiology studies. Large-scale genomic screening revealed a very low prevalence of substitutions affecting *ftsI,* including in resistance isolates, although possible clonal expansion cannot be ruled out. Our findings align with the broader epidemiological landscape in which cefiderocol resistance is heterogeneous and flexible. Therefore, incorporating analysis of *ftsI* variants within or proximal to the active-site into genomic surveillance frameworks could improve resistance detection to this last-resort antibiotic.

**Key points:**

• *Resistance occurred without alterations in iron-uptake systems or siderophore pathways.*

• *Mutations in FtsI (G306V) and AcrR (E91K) were identified as key candidates.*

• *FtsI G306V is rare across cefiderocol resistant and global K. pneumoniae genomes.*

**Supplementary Information:**

The online version contains supplementary material available at https://doi.org/10.1007/s00253-026-13967-y.

## Introduction

The global spread of multidrug-resistant (MDR) Gram-negative bacilli is one of the most critical contemporary infectious diseases challenges worldwide (Potter et al. 2016; Tristancho-Baró et al. 2025a). Carbapenem-resistant Enterobacterales (CRE), particularly carbapenemase-producing *Klebsiella pneumoniae*, are associated with high morbidity and mortality and limited therapeutic options (Xu et al. 2017). In light of this threat, the World Health Organization has classified CRE as critical-priority pathogens, emphasizing the urgent need for research and development of new therapeutic alternatives (2024). Moreover, the increasing prevalence of high-risk clones that co-produce multiple carbapenemases further exacerbates this scenario, often leaving clinicians with only a few salvage treatment options (Chen et al. 2025).

Cefiderocol is a newly developed siderophore-conjugated cephalosporin designed to overcome resistance in MDR Gram-negative bacteria. It has a distinctive structural design that combines key pharmacophoric features of both ceftazidime and cefepime. The C-7 side chain resembles that of ceftazidime, conferring enhanced stability against β-lactamases and activity against non-fermenting Gram-negative bacilli, while the C-3 side chain shares structural similarity with cefepime, contributing to improved permeability and stability. Critically, the addition of a catechol moiety at the C-3 position enables ferric iron chelation, allowing cefiderocol to exploit bacterial iron transport systems for active uptake across the outer membrane via TonB-dependent receptors (Ito et al. 2018). This “Trojan horse” strategy facilitates periplasmic accumulation even in strains with porin loss or outer membrane permeability defects. Once in the periplasmic space, cefiderocol predominantly binds to penicillin-binding protein 3 (PBP3), encoded by *ftsI*, thereby inhibiting peptidoglycan cross-linking, leading to cell wall instability and eventual bacterial death (Wu et al. 2020).

Due to its unique structure and broad enzymatic stability profile, cefiderocol has become a key therapeutic option for treating infections caused by CRE, *Acinetobacter baumannii*, and *Pseudomonas aeruginosa*. Clinical and in vitro studies have demonstrated that cefiderocol exhibits potent activity against CRE, including isolates producing KPC, OXA-48-like, VIM, IMP, and NDM enzymes, and even against strains co-producing multiple carbapenemases, as well as isolates that are resistant to novel β-lactam/β-lactamase inhibitor combinations (Delgado-Valverde et al. 2023). In this context, it has become a last-line therapeutic option for severe infections caused by MDR Gram-negative organisms.

Nevertheless, decreased susceptibility to and resistance against cefiderocol has increasingly been reported, including cases of emergence during therapy. The best-characterized mechanisms involve alterations in iron transport systems and plasmid-mediated determinants affecting ferric citrate transport systems or the expression of β-lactamases variants with altered substrate profiles, particularly KPC and NDM derivatives. Mutations or loss-of-function variants in TonB-dependent siderophore receptors such as CirA and Fiu, as well as alterations in *tonB* and related regulatory systems have been documented in both cefiderocol-exposed and cefiderocol-naïve clinical and isogenic strains, resulting in antibiotic resistance (Karakonstantis et al. 2022).

In contrast, resistance mechanisms directly involving the antibiotic target (i.e., PBP3 inactivation) remain relatively unexplored. Although *ftsI* mutations have been associated with reduced susceptibility to other β-lactams and have occasionally been reported in cefiderocol-resistant isolates, their structural and functional consequences have not been comprehensively characterized (Pasteran et al. 2025). Compared with siderophore receptor alterations, target-mediated resistance through PBP3 modifications has received limited attention, and its contribution to clinically relevant cefiderocol resistance remains poorly defined.

Furthermore, few studies have investigated the emergence of cefiderocol resistance in vivo within a single host using high-resolution comparative genomics. Even fewer investigations have combined hybrid genome assemblies, intra-host variant analysis, structural in silico validation of candidate mutations, and large-scale genomic screening across global datasets in order to contextualize the prevalence and evolutionary distribution of putative resistance determinants.

In this context, we investigated the genomic basis of cefiderocol resistance that emerged during therapy in a clinical isolate of *K. pneumoniae*, using hybrid whole-genome sequencing enabling comparative genomic analysis of paired susceptible and resistant isolates obtained from the same source. Comparative genomic analyses identified candidate mutations in *ftsI* and *acrR*, while structural modelling suggested a potential impact of the FtsI substitution on cefiderocol interactions. Together, these findings provide new insights into candidate mechanisms associated with cefiderocol resistance emergence and highlight the relevance of antimicrobial resistance development under antimicrobial pressure.

## Materials and methods

### Clinical data collection and bacterial isolation and identification

Clinical and microbiological data were retrieved from the institutional Laboratory Information Systems (LIS) (SIGLO®, Horus®) and the electronic medical record. Demographic (i.e., medical diagnosis and antibiotic regimen and duration), microbiological, and antimicrobial treatment data including regimen dose and length were retrospectively reviewed.

The isolates were recovered from intraabdominal collection fluid obtained by aspiration in a sterile environment. Samples were inoculated onto Columbia CNA, chocolate, MacConkey, Sabouraud dextrose, and Schaedler agar plates (Thermo Fisher Scientific®, Waltham, MA, USA) and incubated at 35ºC following standardized laboratory protocols. Identification was performed using matrix-assisted laser desorption ionization time-of-flight mass spectrometry (MALDI-TOF MS).

### Antimicrobial susceptibility testing

Initial antimicrobial susceptibility (AST) testing was performed using a commercial Gram-negative microdilution panel (N57 panel, Beckman Coulter, Inc., Brea, CA, USA) and read using the MicroScan WalkAway® system (Beckman Coulter, Inc., Brea, CA, USA) according to the manufacturer’s instructions. Detection of extended-spectrum β-lactamases (ESBLs) and carbapenemases was carried out using the immunochromatographic assays NG-TEST® CTX-M Multi and NG-TEST® CARBA-5®, respectively (NG Biotech, Guipry-Messac, France), following the manufacturer’s protocols. Cefiderocol susceptibility was determined by broth microdilution using the commercial iron-depleted, cation-adjusted Müeller–Hinton broth-based system ComASP® Cefiderocol (Liofilchem® S.r.l., Roseto degli Abruzzi, Italy), according to the manufacturer’s recommendations. Interpretation was performed according to EUCAST clinical breakpoints v15  (2025).

### Whole-genome sequencing

Prior to genomic DNA extraction, the isolate was subcultured on Columbia blood agar (Thermo Scientific®) plates to increase available biomass. Culture purity was verified through morphological inspection of colonies and confirmatory identification using MALDI-TOF MS.

Genomic DNA for short-read sequencing was extracted using a magnetic bead-based method on the automated MagCore® system (RBC Bioscience Corp., New Taipei City, Taiwan), following the manufacturer’s instructions. Sequencing libraries were prepared using the Nextera™ DNA Library Preparation Kit (Illumina, Inc., San Diego, CA, USA). Libraries were sequenced on an Illumina MiSeq® platform (Illumina, Inc., San Diego, CA, USA) using a MiSeq® Reagent Kit v2 (300 cycles), generating paired-end reads of 150 bp in length.

High-molecular-weight genomic DNA for long-read sequencing was extracted using the MICROBIOME DNA EXTRACTION KIT (Altair Health S.L., Madrid, Spain), according to the manufacturer’s instructions. Sequencing libraries were prepared using the Native Barcoding Kit (Oxford Nanopore Technologies Ltd., Oxford, UK). Libraries were loaded onto a MinION™ device (Oxford Nanopore Technologies Ltd., Oxford, UK) and sequenced for 72 h, following standard Oxford Nanopore Technologies protocols.

### Sequence quality control and preprocessing

Quality assessment of Illumina short reads was performed using FastQC (Andrews 2010) v0.12.1 (Babraham Bioinformatics, Cambridge, UK). Adapter sequences and low-quality bases were removed using Trimmomatic (Bolger et al. 2014) v0.39, applying standard parameters for paired-end trimming (SLIDINGWINDOW:4:20, MINLEN:50). Post-trimming quality metrics were re-evaluated with FastQC to ensure adequate read quality prior to assembly.

Oxford Nanopore Technologies (ONT) reads were assessed using NanoPlot (De Coster and Rademakers 2023) v1.42.0 for evaluation of read length distribution, N50, and quality score metrics. Read filtering and length-based selection and subsampling were performed using Filtlong (Wick https://github.com/rrwick/filtlong accessed 01/10/2025) v0.2.1, prioritizing higher-quality reads for downstream assembly.

### Genome assembly

A long-reads-first hybrid assembly strategy was implemented using Hybracter (Bouras et al. 2024) v0.12.0 with default parameters. Long ONT reads were used as the primary input for initial assembly graph construction, followed by polishing using Illumina short reads to improve base-level accuracy. Circularization of contigs and resolution of replicons were assessed during the assembly process. Plasmid reconstruction was performed using Plassembler (Bouras et al. 2023) (implemented within Hybracter v0.12.0), enabling separation of chromosomal and plasmid components.

Assembly quality metrics, including total genome size, N50, number of contigs, and completeness, were evaluated prior to downstream analyses.

### Genome annotation and genomic characterization

Functional genome quality was assessed using BUSCO (Tegenfeldt et al. 2025) v6.0.0 against the *Enterobacteriaceae* dataset to evaluate completeness based on conserved single-copy orthologs.

Potential contamination was evaluated at two levels. At the raw-read level, taxonomic composition and potential cross-species contamination were assessed using Mash (Ondov et al. 2019) v2.3 by comparing sketch distances against their reference sketch database. At the assembly level, contamination and chimerism were evaluated using GUNC (Orakov et al. 2021) v1.0.1, which detects lineage heterogeneity and possible assembly artifacts based on gene-level taxonomic assignments. Only genomes passing both read-level and assembly-level contamination screening were retained for downstream analyses.

Genomes were annotated using Bakta (Schwengers et al. 2021) v1.11.4 with default parameters and the full database v6.0. Then the resistome was characterized using two complementary approaches: Kleborate (Lam et al. 2021) v3.0.3 for *Klebsiella*-specific resistome, virulome, capsular typing, and sequence type (ST) inference and RGI (Alcock et al. 2023) v6.0.0 (CARD database v4.0.1) for comprehensive antimicrobial resistance gene detection.

Plasmid sequences reconstructed through Plassembler were further characterized using MOB-RECON (Robertson and Nash 2018) v3.1.9 to identify plasmid replicons, mobility groups, and relaxase types.

Multilocus sequence typing (MLST) was inferred using Kleborate v3.0.3 based on the Pasteur scheme.

### Variant calling and comparative genomics

Single nucleotide polymorphisms (SNPs) and small insertions and deletions (indels) were identified using Snippy (Seemann https://github.com/tseemann/snippy accessed 15/10/2025) v4.6.0, which performs read mapping and variant calling using BWA-MEM and FreeBayes internally. Variants were annotated at the locus and amino acid level using SnpEff (Cingolani et al. 2012) v5.2 (implemented within Snippy), based on the reference *K. pneumoniae* genome (see below).

To identify mutations associated with cefiderocol resistance, both the susceptible and resistant isolates were independently mapped against the same reference genome. Variants were filtered using an in-house Python script to retain only those mutations present in the resistant isolate, absent in the susceptible isolate, and resulting in non-synonymous substitutions or predicted functional impact. This approach enabled the identification of de novo mutations emerging during intra-host evolution under cefiderocol selective pressure.

The reference genome used for all comparative analyses was *Klebsiella pneumoniae* subsp. *pneumoniae* HS11286 (BioSample accession SAMN02602959; assembly accession GCF_000240185.1).

As the selected reference sequence may not contain the same plasmid repertoire as the study isolates, plasmid-specific analyses were performed independently. The assembled circularized plasmid sequences from isolate 1268564 were used as references to extract plasmid-derived reads from both isolates and mapped accordingly in order to identify single-nucleotide polymorphisms present in the resistant isolate relative to the susceptible one.

Structural variation was assessed by nucleotide identity comparisons using BLAST (McGinnis and Madden 2004) and fastANI (Jain et al. 2018), while gene gain or loss events were evaluated through comparative analysis of the annotated plasmid sequences from both isolates.

### Structural modelling and comparative analysis of the PBP3 G306V variant

#### Selection of the structural template

A three-dimensional structural model of *K. pneumoniae* PBP3 (FtsI) was generated using the AlphaFold-predicted structure (UniProt ID A0A0H3GN85). The reference structure was selected based on its taxonomic match to *K. pneumoniae*, resolution < 2.5 Å, completeness of the transpeptidase domain, and absence of major backbone gaps in the region encompassing residues 300–310. The catalytic serine and surrounding active-site architecture were manually inspected to ensure structural integrity prior to in silico mutagenesis. The selected structure was prepared by removing non-protein heteroatoms, adding missing side-chains where necessary, and standardizing residue numbering to match the *K. pneumoniae* reference sequence used for variant calling (i.e., strain HS11286).

#### In silico mutagenesis and stability analysis

The FtsI G306V substitution was introduced using FoldX (Schymkowitz et al. 2005; Delgado et al. 2019) v5.0 after repairing the input structure with the RepairPDB function. To minimize rotamer bias, five independent BuildModel replicates were performed for both wildtype (WT) and mutant (MUT) structures under identical conditions. For each replicate, the folding free energy (ΔG) was calculated, and the mean ΔG for WT and MUT was obtained. The stability difference was calculated as ΔΔG = ΔGMUT − ΔGWT. A ΔΔG greater than 1.6 kcal/mol was interpreted as probable destabilizing relative to the WT structure (Schymkowitz et al. 2005).

#### Structural visualization and geometric measurements

Structural analyses were performed in PyMOL (Schrödinger, LLC. The PyMOL Molecular Graphics System) v3.1.4. WT and MUT models were aligned using backbone atoms of the transpeptidase domain. Visualization focused on residues 305–310. Distances were measured using the *dist* command between: Ser307 Oγ (OG) and the β-lactam carbonyl carbon (C_lactam), Ser307 Oγ and the β-lactam carbonyl oxygen (O_lactam), CA306–OG307 (structural reference), CB306–OG307 (MUT only), CA306–CG1 (valine side-chain extension). Distances were calculated in Ångström and reported as mean ± SD where applicable.

#### Geometric evaluation

The three-dimensional structure of cefiderocol was obtained from the PubChem database (CID 77843966). The ligand was geometry-optimized and converted to PDBQT format using Open babel (O’Boyle et al. 2011) v3.1.1 and MGLTools (Forli et al. 2016) v1.5.7. The β-lactam carbonyl carbon and oxygen were manually identified based on chemical structure and verified against crystallographic data reported for PBP3-cefiderocol complexes elsewhere. The structural orientation of the β-lactam carbonyl was cross-checked with published crystallographic data describing acyl-enzyme complexes of PBP3 to ensure correct atom assignment. To assess compatibility with catalytic acylation, the following geometric parameters were measured: Nucleophile–electrophile distance d(Oγ–C_lactam), Bürgi–Dunitz attack angle between: Oγ (Ser307), C_lactam and O_lactam. An optimal Bürgi–Dunitz trajectory is expected at ~ 105–110°. Angles were measured using the PyMOL *angle* function. Chi1 torsional angles (N–CA–CB–OG) and CA–CB–OG bond angles were also evaluated to detect conformational rearrangements of the catalytic serine.

#### Molecular docking

Docking simulations were performed using Vina (Eberhardt et al. 2021) v1.2.6. The receptor was converted to PDBQT format after addition of polar hydrogens and assignment of Gasteiger charges. The search space was centered on the catalytic serine (i.e., S307) and encompassed the active-site cavity using a grid box defined by center_x (−15.11400032043457), center_y (−5.921000003814697), and center_z (16.163000106811523) (Å) as well as size_x (24), size_y (24) and size_z (24) (Å). Exhaustiveness was set to 8, and up to 30 binding modes were generated per run. Docking was performed independently for WT and MUT models under identical parameters. All ranking poses were visually inspected to confirm positioning within the active-site and perform measurements.

#### Post-docking geometric filtering

For each docking pose, distances between Ser307 Oγ and C_lactam and O_lactam were measured. A pose was considered geometrically compatible with nucleophilic attack if d(Oγ–C_lactam) ≤ 3.5 Å and d(Oγ–Olactam) ≤ 3.5 Å. The number of poses meeting this criterion was counted for WT and MUT.

### Selection of publicly available cefiderocol-resistant genomes and SNP calculation

A systematic literature search was performed up to December 31, 2025, using the following PubMed query “cefiderocol[Title]”. Publications were screened for studies reporting *K. pneumoniae* isolates with phenotypically confirmed cefiderocol resistance. Studies were included when cefiderocol susceptibility data were provided and genome sequences were deposited in publicly accessible repositories (e.g., NCBI, ENA). Only isolates meeting both criteria were selected for downstream genomic and phylogenomic analyses. SNP profile of publicly available cefiderocol-resistant genomes was generated using the same workflow as described above (Snippy v4.6.0 and SnpEff v5.2).

### Phylogenomic analysis

Raw sequencing reads from selected publicly available genomes were assembled using Unicycler (Wick et al. 2017) v0.5.1 under default hybrid or short-read mode, depending on available data. Core genome alignment was performed using Parsnp (Kille et al. 2024) v1.7.4, aligning assembled genomes against a reference chromosome (i.e., strain HS11286). The resulting core genome alignment was used as input for phylogenetic inference using IQ-TREE (Hoang et al. 2018; Minh et al. 2020) v2.3.5. Maximum likelihood phylogeny was reconstructed under the GTR + F + G4 substitution model. Branch support was assessed using 1000 ultrafast bootstrap replicates and 1000 SH-aLRT replicates. Phylogenetic tree was visualized and annotated using iTOL (Letunic and Bork 2024) v6.

### Global screening across complete *K. pneumoniae* genomes

Chromosome-level closed genomes of all *K. pneumoniae* available in RefSeq up to October 31, 2025, were downloaded and annotated as described above. Protein sequences corresponding to candidate loci were extracted from the proteomes using an in-house script and aligned using MAFFT (Katoh and Standley 2013) v7.525 under the L-INS-i strategy for high-accuracy. Aligned sequences exhibiting residues different from Gly at position 306 for FtsI and Glu at position 91 for AcrR were extracted using an in-house script and manually inspected to determine whether amino acid residues at the positions of interest differed from the wild-type sequence identified in the susceptible isolate.

### Other statistical analyses

Descriptive and inferential statistical analyses were performed in R v4.5.1 using ggstatsplot v0.12.0 and rstatix v0.7.2. A two-tailed *p*-value < 0.05 was considered statistically significant.

## Results

Isolate 1268564 was first recovered on hospital day 70 (18/08/2024) from purulent material obtained by percutaneous drainage of an intra-abdominal collection. The patient had a prior history of Myasthenia gravis and had been admitted for necrohemorrhagic pancreatitis complicated by extensive intra-abdominal collections that required prolonged hospitalization, repeated drainage procedures, and sustained exposure to multiple antimicrobial regimens.

Management was particularly challenging due to the difficulty in achieving definitive source control and the polymicrobial nature of the infection, with sequential isolation of several MDR organisms from drainage samples (i.e., Vancomycin-resistant *Enterococcus faecium*, carbapenem-resistant non-carbapenemase-producing *Pseudomonas aeruginosa*, and a VIM-1-producing *Escherichia coli)*. In addition, Myasthenia gravis prevents the use of some antimicrobials. Consequently, antimicrobial therapy was repeatedly reassessed and adapted according to the patient’s clinical course and microbiological findings.

**Fig. 1:**
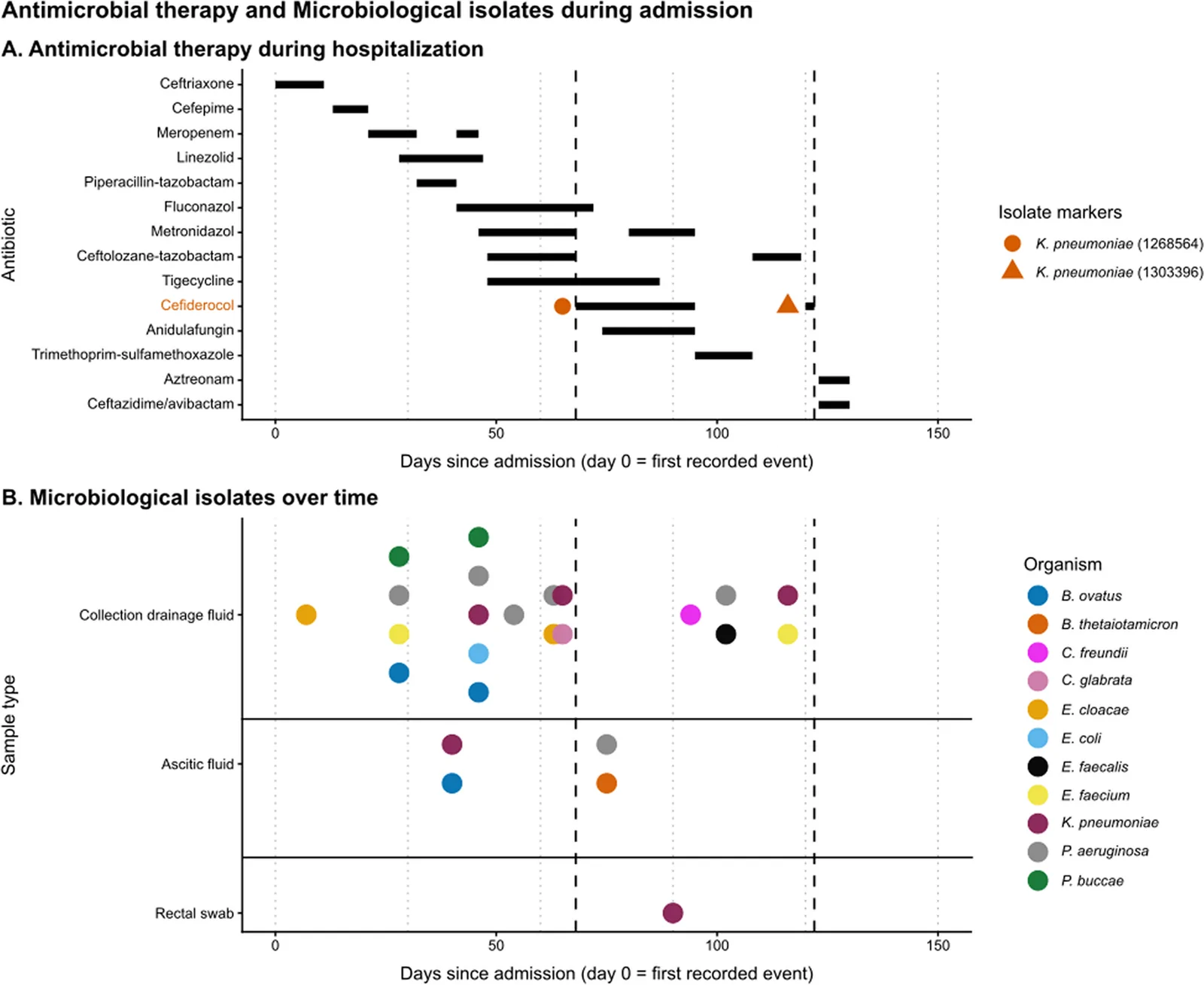
Antimicrobial therapy and microbiological isolates during hospitalization. **A** The antimicrobial regimens administered during hospitalization are shown, including their duration and the temporal relationship with the emergence of isolates 1268564 and 1303396. Antimicrobial therapy was sequentially adapted according to clinical evolution, source control procedures, and microbiological results. Light dashed vertical lines indicate 30-day intervals. Dark dashed bars denote the period between the first and last dose of cefiderocol. **B** The temporal evolution of microbiological isolates during hospitalization is displayed

Isolate 1303396 was subsequently recovered from the same patient on hospital day 128, following 27 consecutive days of cefiderocol therapy that had been initiated for a persistent intra-abdominal infection caused by MDR Gram-negative bacilli, including VIM-1-producing *E. coli*, VIM-1-producing *K. pneumoniae*, and difficult-to-treat *P. aeruginosa* as illustrated in Fig. 1. After 24 h of incubation, growth was observed on chocolate and MacConkey agar. Colonies were identified as *K. pneumoniae* by MALDI-TOF MS. Immunochromatographic assays confirmed the absence of CTX-M-type extended-spectrum β-lactamase and presence of VIM type carbapenemase in both isolates.

AST demonstrated that isolate 1268564 exhibited an extensively drug-resistant phenotype (Table 1), displaying resistance to all tested β-lactams with the exception of aztreonam. It showed also resistance to fluoroquinolones, trimethoprim/sulfamethoxazole and tobramycin, but remained susceptible to gentamicin, amikacin, and colistin. Cefiderocol testing yielded a minimum inhibitory concentration of 1 mg/L, which corresponded to susceptibility . In contrast, isolate 1303396 regained susceptibility to carbapenems with MICs of 0.5, 2, and 1 mg/L for ertapenem, imipenem, and meropenem, respectively, as well as susceptibility to tobramycin compared to 1268564. These results were verified using both the broth microdilution and gradient strip methods. Regarding the cefiderocol profile, the minimum inhibitory concentration increased from 1 to 32 mg/L, classifying the isolate as resistant.

**Table 1:** Antibiotic susceptibility testing results of both isolates

Antimicrobial	1268564 MIC (mg/L)	Category	1303396 MIC (mg/L)	Category
Piperacillin/tazobactam	> 16	R	> 16	R
Cefotaxime	> 32	R	> 32	R
Ceftazidime	> 32	R	> 32	R
Cefepime	> 8	R	> 8	R
Aztreonam	≤ 1	S	4	SIE
Ertapenem	> 1	R	0.5	S
Imipenem	> 4	R	2	S
Meropenem	> 8	R	1	S
Ciprofloxacin	> 1	R	> 1	R
Levofloxacin	> 1	R	> 1	R
Gentamycin	≤ 2	S	≤ 2	S
Tobramycin	4	R	≤ 2	S
Amikacin	≤ 8	S	≤ 8	S
Trimethoprim/sulfamethoxazole	> 4/76	R	> 4/76	R
Colistin	≤ 2	S	≤ 2	S
Ceftazidime/avibactam	> 8	R	> 8	R
Meropenem/vaborbactam	> 8	R	2	S
Ceftolozane/tazobactam	> 4	R	> 4	R
Cefiderocol	1	S	32	R

Presents the MICs for each antibiotic tested in both isolates. The corresponding clinical category according to EUCAST v15 criteria is indicated. R=Resistant, S=Susceptible, SIE=Susceptible increased exposure

Whole-genome sequencing produced high-quality datasets for both isolates. Following quality filtering, Illumina sequencing achieved a depth of coverage > 200X, with over 99% of reads exhibiting quality scores of at least Q35. Long-read sequencing achieved a depth of coverage of approximately 80X for isolate 1268564 and 107X for isolate 1303396, with a median read quality score of Q17. Using a long-reads-first strategy for hybrid assembly allowed the reconstruction of a single circular chromosome of 5,438,344 base pairs, with a guanine-cytosine content of 57.35% in both isolates, together with five fully circularized plasmids. Functional completeness was high, with 99.1% of conserved *Enterobacteriaceae* single-copy orthologs detected, and no evidence of contamination was identified at either the read or assembly level. Genome annotation identified 5584 coding sequences, 94 transfer RNA genes, 24 ribosomal RNA operons, 82 non-coding RNAs, 1 transfer–messenger RNA, 2 CRISPR arrays, and 4 replication origins, comprising one chromosomal origin and three plasmid-associated vegetative replication origins. Only 160 proteins remained annotated as hypothetical, and seven as pseudogenes, supporting the high-quality of the assembly and annotation.

Both isolates belonged to sequence type (ST) 14 within the *K. pneumoniae* species complex and were predicted as capsular type K2 and O-antigen type O2α. They carried a yersiniabactin siderophore system with an ST 5, encoded within the integrative conjugative element ICEKp6, while colibactin, aerobactin, salmochelin, and the rmpADC loci were absent, consistent with a kaptive virulence score of 1.

Resistome analysis demonstrated that both isolates shared an identical set of acquired and chromosomally encoded resistance determinants. The resistome was characterized by the presence of *bla*_*VIM-1*_, *bla*_*SHV-1*_, and *bla*_*SHV-28*_, located on plasmid 4, plasmid 1, and the chromosome, respectively. In addition, plasmid 4 harbored the aminoglycoside resistance gene *aadA1*, while *qnrS1* was identified on plasmid 2, accounting for the elevated MICs observed for tobramycin and fluoroquinolones. Furthermore, chromosomally encoded active efflux systems were detected, most notably *kpnEFG*, *oqxAB*, and *acrAB*. The complete inferred resistome of isolate 1303396 is presented in Table 2.

**Table 2:** Main resistome of isolate 1303396

Sample	ST	K/O Type	Betalactams	Fluoroquinolones	Aminoglycosides	Efflux	Others
1303396	14	2/O2α	VIM-1; SHV-28; SHV-1	qnrS1; aac(6')	aadA1; aac(6')	kpnFGH; oqxAB; acrABD;	sul1; mphE; dfrB1; catB2

Summarizes the principal findings of strain typing and resistome characterization of isolate 1303396. ST stands for Sequence type

The plasmidome of both isolates consisted of five plasmids ranging in size from 8302 to 188,299 bp (Supplementary Table 1). Among these, two were classified as conjugative, one as mobilizable, and two as non-mobilizable based on the presence or absence of complete transfer and mobilization machinery. The carbapenemase gene *bla*_*VIM-1*_ was located on a conjugative plasmid belonging to the IncL/M incompatibility group. This plasmid encoded a complete mating pair formation system type I together with MOBP relaxase machinery, supporting its classification as conjugative. The *bla*_*VIM-1*_ gene was embedded within a composite transposon structure flanked by three copies of IS26 and one IS1SD insertion element, consistent with a mobilizable resistance platform. The same plasmid also carried the aminoglycoside resistance determinant *aadA1*.

A second conjugative plasmid belonged to the IncFII incompatibility group and encoded MOBF relaxase genes and a complete mating pair formation system type F. This replicon harboured *bla*_*SHV-1*_*,* alongside accessory antimicrobial resistance determinants. In contrast, *bla*_*SHV-28*_ was located on the chromosome, consistent with the intrinsic SHV lineage frequently observed in ST 14 isolates. A third plasmid carried *qnrS1* and encoded mobilization functions but lacked a complete transfer machinery, indicating its dependence on co-resident conjugative plasmids for dissemination. Two additional small plasmids lacked identifiable conjugative or mobilization systems containing predominantly maintenance genes and hypothetical open reading frames.

Comparative analysis demonstrated a nucleotide identity greater than 99.99% across all plasmid replicons between isolates, indicating that no plasmid acquisition, loss, or major structural rearrangement occurred during therapy. A minor accessory region of approximately 2.6 kb was identified on the largest plasmid of isolate 1303396, which encodes no antibiotic resistance mechanisms; however, this region consisted exclusively of hypothetical proteins and reannotated pseudogenes and did not include resistance or iron-uptake determinants. At the same time, four SNPs were present in the *aadA1* locus (i.e. the plasmid containing the *bla*_VIM-1_ locus). This precludes the possibility that extrachromosomal genetic material was responsible for the phenotypic shift in antimicrobial susceptibility observed between the two isolates. Overall, plasmid architecture remained highly conserved, with variation restricted to a localized mobile element insertion and the *aadA1* locus. No differences were observed in siderophore-related loci, iron transport systems, or outer membrane porin genes between the plasmids of both isolates. Pairwise sequence comparison of all β-lactamase genes, including *bla*_VIM-1_, *bla*_SHV-1_, and *bla*_SHV-28_ was conducted. This analysis demonstrated 100% nucleotide identity between the corresponding genes of both isolates.

Chromosomal comparison identified 82 SNPs in isolate 1303396 relative to isolate 1268564, comprising 41 missense substitutions, 35 synonymous substitutions, 2 nonsense mutations, 2 frameshift mutations, and 2 complex variants. Most missense substitutions affected genes involved in transport functions, replication-associated proteins, or hypothetical coding sequences, most of them of plasmid origin. Additionally, four missense substitutions were detected within *aadA1*. Among all chromosomal variants, only two were considered biologically plausible candidates given the known biological functions of the encoded proteins and their potential relevance to antimicrobial resistance mechanisms: *ftsI*:917G > T and *acrR*:271G > A, resulting in G306V and E91K amino acid substitutions, respectively. Biological plausibility was inferred due to their respective roles in peptidoglycan biosynthesis and the extrusion of compounds from the cell through transcriptional regulation of the AcrAB efflux system. The full set of variants found between 1268564 and 1303396 is shown in Table 3. Importantly, *ftsI*:917G > T was supported by a covering depth of 252X.

**Table 3:** Differences of SNP profile between 1303396 and 1268564

POS	Type	REF	ALT	Evidence	Strand	NT_POS	AA_POS	Effect
937	complex	GTT	AAA	AAA:30 GTT:0	+	215/417	72/138	Missense
1327	snp	T	C	C:44 T:0	-	678/780	226/259	Synonymous
2338	snp	G	C	C:36 G:0	-	855/1005	285/334	Synonymous
2344	complex	AAC	CAG	CAG:37 AAC:0	-	849/1005	283/334	Missense
2351	complex	TTTTC	CTTTT	CTTTT:45 TTTTC:0	-	842/1005	280/334	Missense
4263	snp	A	G	G:33 A:0	-	180/393	60/130	Synonymous
4269	snp	C	T	T:39 C:0	-	174/393	58/130	Synonymous
4278	complex	GCGG	ACGA	ACGA:41 GCGG:0	-	165/393	54/130	Synonymous
4296	snp	G	C	C:50 G:0	-	147/393	49/130	Synonymous
4308	complex	GTCAGT	ATCGGA	ATCGGA:53 GTCAGT:0	-	135/393	44/130	Missense
4324	snp	C	G	G:54 C:0	-	119/393	40/130	Missense
4331	snp	C	T	T:54 C:0	-	112/393	38/130	Missense
4360	snp	G	T	T:51 G:0	-	83/393	28/130	Missense
4372	snp	C	T	T:46 C:0	-	71/393	24/130	Missense
4377	snp	T	C	C:40 T:0	-	66/393	22/130	Missense
4385	complex	GGAAG	TGAAA	TGAAA:37 GGAAG:0	-	58/393	18/130	Missense
4394	snp	C	T	T:33 C:0	-	49/393	17/130	Missense
5704	snp	A	G	G:39 A:0	-	342/462	114/153	Synonymous
5710	snp	G	C	C:55 G:0	-	336/462	112/153	Synonymous
20,754	complex	GCAGA	ACAGG	ACAGG:34 GCAGA:0	-	307/411	101/136	Synonymous
21,232	complex	CGGCTGATCGA	TGGCAGTTCGG	TGGCAGTTCGG:38 CGGCTGATCGA:0	-	444/606	145/201	Missense
39,829	snp	T	C	C:50 T:0	-	419/480	140/159	Missense
40,325	snp	T	C	C:37 T:0	-	711/780	237/259	Synonymous
40,996	complex	TTAGC	CAACT	CAACT:39 TTAGC:0	-	40/780	12/259	Missense
41,015	complex	GT	AG	AG:31 GT:0	-	21/780	7/259	Missense
41,021	complex	AAT	TAC	TAC:31 AAT:0	-	15/780	5/259	Missense
41,028	complex	ATGG	GCGA	GCGA:31 ATGG:0	-	8/780	2/259	Missense
45,970	snp	C	G	G:33 C:0	-	705/726	235/241	Synonymous
48,683	snp	C	G	G:73 C:0	-	3330/5259	1110/1752	Synonymous
48,691	complex	AA	CG	CG:48 AA:0	-	3322/5259	1107/1752	Missense
48,697	snp	A	G	G:47 A:0	-	3316/5259	1106/1752	Missense
48,800	complex	ATCA	GTCG	GTCG:36 ATCA:0	-	3213/5259	1070/1752	Synonymous
48,815	snp	C	G	G:69 C:0	-	3198/5259	1066/1752	Synonymous
50,674	complex	AGCG	CGCA	CGCA:30 AGCG:0	-	2010/2622	669/873	Synonymous
52,245	complex	CA	TT	TT:73 CA:0	-	2047/2280	682/759	Missense
52,254	snp	T	C	C:61 T:0	-	2038/2280	680/759	Missense
52,260	complex	CAGAT	AAGAG	AAGAG:53 CAGAT:0	-	2032/2280	676/759	Missense
52,275	snp	C	A	A:30 C:2	-	2017/2280	673/759	Missense
52,366	ins	C	CT	CT:37 C:0	-	1925/2280	642/759	Frameshift
52,376	complex	GCA	TCC	TCC:46 GCA:0	-	1916/2280	638/759	Missense
66,899	complex	AAAA	GAAG	GAAG:40 AAAA:0	-	2442/3201	813/1066	Synonymous
67,397	snp	T	C	C:40 T:0	-	1944/3201	648/1066	Synonymous
73,596	ins	C	CG	CG:41 C:0	+	83/126	28/41	Frameshift
105,208	snp	G	T	T:34 G:0	-	246/594	82/197	Synonymous
119,743	complex	GATAA	AATAG	AATAG:44 GATAA:0	+	117/117	39/38	Complex
355,636	complex	AAATTTGA	GAATATGG	GAATATGG:39 AAATTTGA:0	+	858/1098	286/365	Missense
419,054	snp	G	C	C:34 G:0	-	85/864	29/287	Missense
419,067	snp	T	C	C:48 T:0	-	72/864	24/287	Synonymous
419,322	snp	A	C	C:34 A:0	-	1953/2124	651/707	Synonymous
419,637	complex	GGTGGGCGACAACAG	AGTAGGTGAAAGCAA	AGTAGGTGAAAGCAA:30 GGTGGGCGACAACAG:0	-	1638/2124	542/707	Synonymous
420,126	snp	G	A	A:37 G:0	-	1149/2124	383/707	Synonymous
427,643	snp	A	T	T:30 A:0	-	339/687	113/228	Synonymous
427,655	snp	T	C	C:33 T:0	-	327/687	109/228	Synonymous
427,661	snp	C	G	G:32 C:0	-	321/687	107/228	Synonymous
427,691	snp	A	G	G:35 A:0	-	291/687	97/228	Synonymous
428,429	snp	G	A	A:37 G:0	-	174/564	58/187	Synonymous
594,233	complex	GATCCGATTCACT	AGCCCGATTCACC	AGCCCGATTCACC:30 GATCCGATTCACT:0	+	9/267	mar-88	Missense
853,042	snp	G	T	T:252 G:0	+	917/1767	306/588	Missense
1,242,483	snp	G	T	T:219 G:0	-	1855/3147	619/1048	Missense
1,245,966	snp	G	A	A:228 G:0	+	271/651	91/216	Missense
1,289,790	snp	A	G	G:35 A:0	-	177/216	59/71	Synonymous
1,436,685	complex	CTGCTTT	ATGTTTC	ATGTTTC:180 CTGCTTT:0	-	426/654	140/217	Missense
1,917,814	complex	CGCACG	TGCGCT	TGCGCT:30 CGCACG:0	-	750/774	249/257	Missense
1,944,995	snp	G	A	A:40 G:0	+	387/414	129/137	Synonymous
2,882,664	snp	G	C	C:169 G:0	-	636/1062	212/353	Missense
2,908,781	complex	TGGCAAG	CGGCAAA	CGGCAAA:39 TGGCAAG:0	+	222/324	74/107	Synonymous
3,242,376	complex	GTGAGCTGCTCTTCC	TTCAATTGCCGCTGA	TTCAATTGCCGCTGA:30 GTGAGCTGCTCTTCC:0	-	1439/3411	475/1136	Missense
3,242,396	complex	ATGC	TTCA	TTCA:28 ATGC:0	-	1419/3411	472/1136	Missense
3,250,514	snp	A	T	T:74 A:6	-	25/894	9/297	Missense
3,251,169	snp	T	C	C:42 T:0	-	447/894	149/297	Synonymous
3,620,052	snp	C	A	A:255 C:0	-	322/1464	108/487	Missense
3,703,860	snp	G	T	T:264 G:0	+	1050/1590	350/529	Missense
4,391,775	complex	TCAT	ATCAC	ATCAC:177 TCAT:0	-	4/114	ene-37	Nonsense
4,414,363	snp	G	C	C:30 G:0	+	1017/1020	339/339	Synonymous
4,682,631	snp	C	A	A:226 C:0	+	78/777	26/258	Nonsense
4,682,783	snp	C	T	T:208 C:0	+	230/777	77/258	Missense
4,758,409	snp	G	C	C:241 G:0	-	579/849	193/282	Synonymous
4,764,954	snp	A	C	C:240 A:0	+	1369/1434	457/477	Missense
4,923,179	snp	T	A	A:232 T:0	-	458/1356	153/451	Missense
4,946,628	snp	A	G	G:30 A:0	+	1491/1509	497/502	Synonymous
5,124,170	snp	A	G	G:34 A:0	-	1032/1137	344/378	Synonymous
5,283,839	ins	G	GGCGCAT	GGCGCAT:183 G:14	-	1117/1221	373/406	Complex

Presents all nucleotide variants identified between isolates 1268564 and 1303396, including their genomic location and predicted functional effect. POS=Genomic position in the reference, REF=Nucleotide present in the reference, ALT=Alternative allele found in the sample, NT_POS=Nucleotide position, AA_POS=amino acid position

**Fig. 2:**
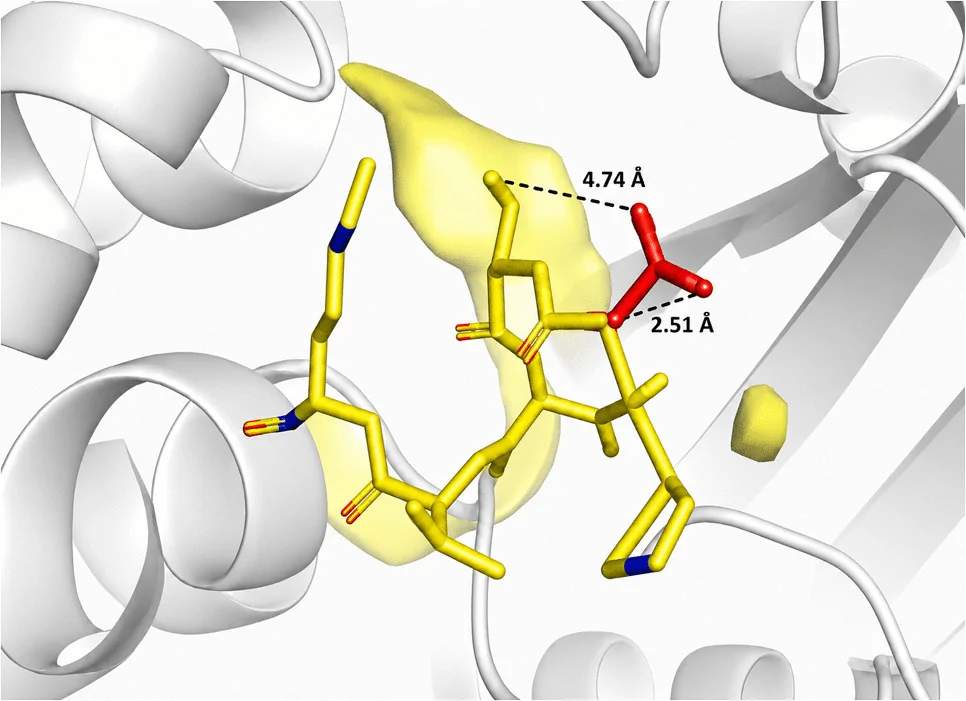
Three-dimensional representation of the region proximal to the active-site. The figure shows the three-dimensional representation of the region proximal to the active-site, specifically residues S307 and V306 in the mutated FtsI model. The side chains are depicted as yellow tubular structures, with the V306 side-chain highlighted in red. Dashed lines indicate the measured interatomic distances between the structures. Distances are expressed in angstroms

In silico mutagenesis of FtsI involved the introduction of the G306V substitution in the three-dimensional model (UniProt ID A0A0H3GN85) of HS11286 reference strain, and its three-dimensional and stability analysis. Three-dimensional and stability analysis predicted a destabilizing effect with a mean ΔΔG of + 6.19 kcal/mol, primarily due to an increase in steric clashes, which was partially offset by an increase in hydrophobic solvation. Structural modelling of the G306V substitution revealed the introduction of a bulky isopropyl side chain at position 306. This substitution of glycine by valine resulted in the emergence of additional side-chain atoms Cβ, Cγ1 and Cγ2, that were absent in the wild-type structure. In the MUT model, the distance between Cβ306 and Oγ307 measured 4.74 Å. Although the WT model lacks Cβ atom for reference, this is consistent with the increment in steric clashes found in the stability analysis. Moreover, the Cα306–Cγ1 intra-residue distance of 2.51 Å, consistent with the expected geometry of a valine side-chain, accounted for further steric forces. Figure 2 illustrates the three-dimensional structural model and the measured interatomic distances between the side-chain atoms of V306 and S307 in the MUT model.

Noteworthy, S307 underwent a conformational adjustment in the MUT model, with the Cα–Cβ–Oγ angle increasing from 109.59 to 124.35° and the χ1 dihedral angle shifting from − 70.18 to − 55.21°. This indicates localized side-chain reorientation without backbone displacement.

Docking simulations demonstrated that 93 percent of WT models adopted geometries compatible with conformations for effective acylation (Supplementary Table 5), defined as a distance of 3.5 Å or less between the catalytic Ser307 Oγ and the β-lactam carbonyl (and its oxygen) of cefiderocol, whereas only 10 percent of MUT models fulfilled this criterion (difference of 83%; 95% CI 0.56–0.90 *p* < 0.05). This significant reduction could modulate the formation of an acyl-enzyme intermediate in the G306V variant (Flanders et al. 2022; Radojković et al. 2025).

The presence of the FtsI G306V mutation was investigated in a cohort of cefiderocol-resistant *K. pneumoniae* genomes in order to assess its prevalence in a different background. To ensure a comprehensive search, all PubMed publications containing the descriptor “cefiderocol” in the title were screened. Of the 625 titles retrieved, 34 reported genomic data from *K. pneumoniae* isolates alongside antimicrobial susceptibility information, and 28 provided access to publicly available sequencing data. After duplicate entries were removed and species identification confirmed, 184 genomes were included in the analysis. These isolates were collected between 2015 and 2025 and originated from eight distinct geographical regions.

The presence of variants in *ftsI* and *acrR* was investigated within this dataset using the same analytical pipeline that was applied to the study isolates. A total of 889 variants were identified in *ftsI* and 135 in *acrR*. Of these, three and eight were classified as missense variants in *ftsI* and *acrR*, respectively, including one isolate harbouring a double substitution in *acrR*. None of the detected variants corresponded to those identified in isolate 1303396. The complete set of missense variants identified among cefiderocol-resistant isolates is provided in Supplementary Table 2.

**Fig. 3:**
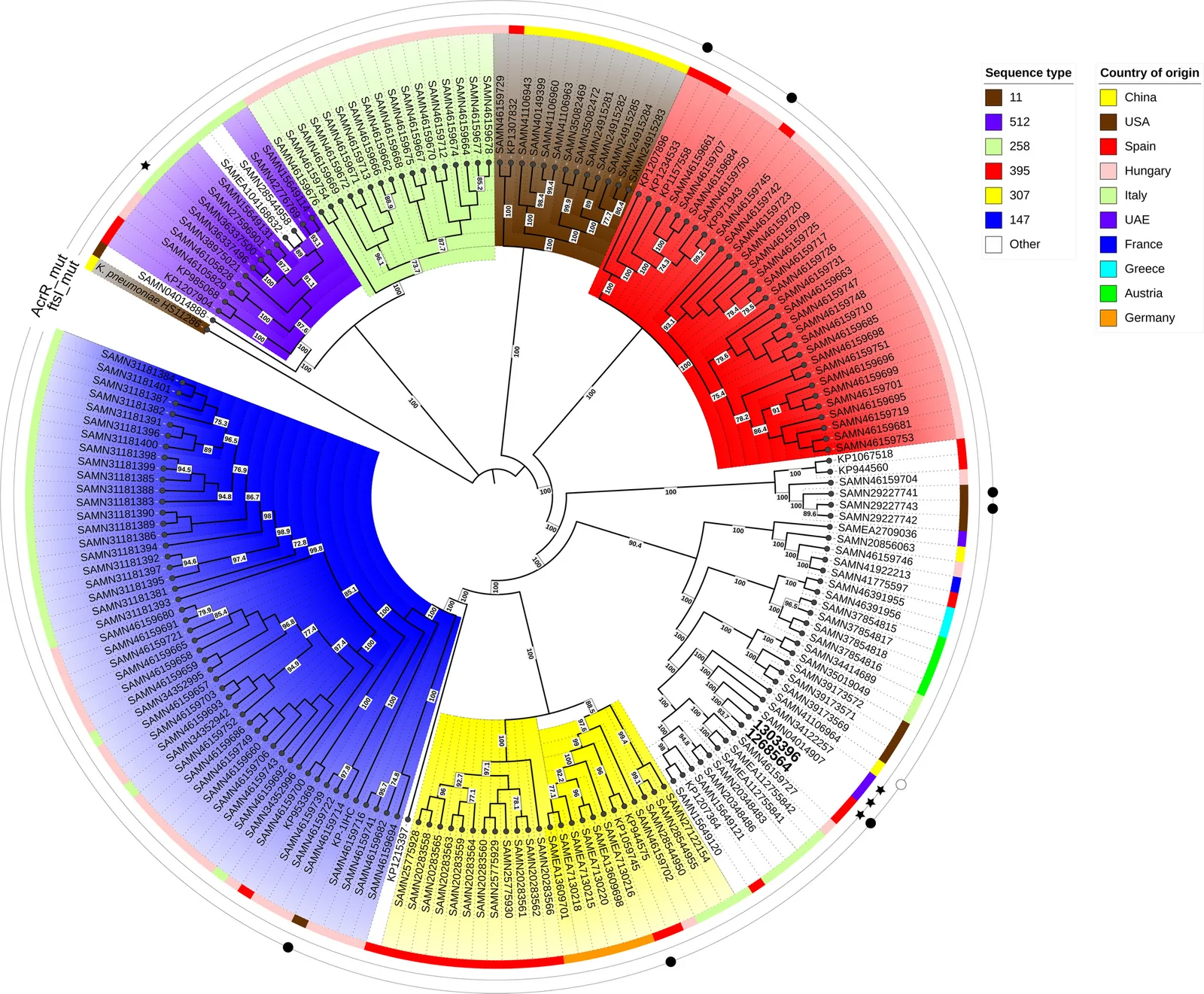
Phylogenetic relationships among cefiderocol-resistant isolates. The figure illustrates the phylogenetic relationships among cefiderocol-resistant isolates identified in the literature. Clade shading corresponds to sequence type (ST). The concentric bars, from the innermost to the outermost layer, represent the country of origin, the presence of mutations in FtsI (indicated by a star) and the presence of mutations in AcrR (indicated by a circle). Bootstrap support values greater than 70 are displayed within nodes (white-background)

To contextualize the phylogenetic placement of isolates 1268564 and 1303396 within the resistant population, core genome single-nucleotide polymorphism analysis was performed. Notably, two of the three isolates harbouring a missense mutation in *ftsI*, corresponding to BioSample accessions SAMN34122257 and SAMN04014907, clustered phylogenetically with 1268564 and 1303396 despite the former isolation dates corresponded to 2021 and 2015, respectively. Both isolates originated from the United Arab Emirates and also belonged to ST 14. However, their resistome profiles differed from that of 1303396. One isolate carried a dual carbapenemase combination consisting of *bla*_*NDM-1*_ and *bla*_*OXA-232*_, whereas the other lacked acquired carbapenemase genes. Their respective cefiderocol MICs were 256 and 8 mg/L. The phylogenomic relationship of the isolates, together with their geographic origin, sequence type, and the presence of mutations in *ftsI* and *acrR*, is shown in Fig. 3. Provenance of resistant isolates is presented in the Supplementary Table 3.

Finally, to provide a broader epidemiological context for the variants identified in this study, we investigated the presence of FtsI G306V and AcrR E91K across all complete and closed *K. pneumoniae* genomes deposited in the NCBI RefSeq database. A total of 2834 genomes were reannotated using a standardized reference to ensure positional comparability. The predicted FtsI and AcrR protein sequences were extracted from each proteome, aligned, and screened for amino acid substitutions. No evidence of either FtsI G306V or AcrR E91K was detected in any of the analysed genomes, supporting the low prevalence of these variants in the global population structure of *K. pneumoniae*.

## Discussion

The present study provides a detailed genomic and structural characterization of a cefiderocol-resistant *K. pneumoniae* strain emerging in vivo during prolonged therapy. By analysing a paired susceptible-resistant isolates recovered before and after 27 consecutive days of cefiderocol exposure, our results suggest that cefiderocol resistance may arise through mechanisms different from the most frequently described in the literature related to iron-uptake disruptions (Klein et al. 2022; Karakonstantis et al. 2022; Lan et al. 2022; Kriz et al. 2024; Long et al. 2025); instead implicating a target-site alteration in PBP3 as a working hypothesis. These findings broaden the current understanding of cefiderocol resistance and highlight the wide flexibility of this phenotype, particularly under sustained selective pressure.

Cefiderocol was designed to overcome classical β-lactam resistance by coupling siderophore-mediated active uptake with stability against most β-lactamases, including carbapenemases (Sato and Yamawaki 2019). Accordingly, the majority of reported resistance mechanisms in Enterobacterales have focused on impaired iron transport, particularly loss-of-function mutations in TonB-dependent receptors such as CirA and Fiu, alterations in TonB/ExbB/ExbD complexes, or regulatory changes affecting iron acquisition (Lan et al. 2022; Moon and Huang 2023; Polani et al. 2025). In vitro evolution experiments consistently identify *tonB* and *cirA* as recurrent adaptive targets, and clinical reports of resistance emerging during therapy in NDM, VIM, and KPC-producing *K. pneumoniae* frequently describe additional truncation or inactivation of siderophore receptors (Zhang et al. 2022; Simner et al. 2022; Deroche et al. 2025). Large-scale genomic analyses have further demonstrated associations between incomplete CirA proteins and elevated cefiderocol MICs in certain high-risk clones (Klein et al. 2022).

In contrast, our paired isolates did not exhibit differential disruption of siderophore receptor genes, TonB systems, or other iron-uptake components. Plasmid architecture remained conserved, and there was no acquisition of ferric citrate transport systems (i.e., *fec* operon) or novel β-lactamase variants. This is particularly relevant given recent data demonstrating that plasmid-located ferric citrate transport can modulate cefiderocol susceptibility in specific KPC-producing backgrounds through iron-dependent regulatory effects (Polani et al. 2025). The absence of such changes in our isolates argues against an influx-mediated mechanism and shifts attention towards other possibilities such as chromosomal target-level adaptation.

Although a total of 82 variants differentiated the susceptible and resistant isolates (Table 3), most affected missense variants (41 in total) outside the selected candidates affected hypothetical proteins, plasmid conjugation proteins, nuclear-associated cellular functions, or other housekeeping functions with no apparent connection to the known mechanism of action of cefiderocol or previously described resistance pathways (Supplementary Table 4). In contrast, the variants identified in *ftsI* and *acrR* affected proteins with established roles in β-lactam activity and antimicrobial susceptibility, making them biologically plausible candidates for further investigation. Nevertheless, the contribution of additional variants cannot be completely excluded. Resistance phenotypes often emerge from complex adaptive processes involving multiple genetic changes, and epistatic interactions between mutations may influence the magnitude or even the direction of their phenotypic effects. Consequently, while the available genomic and structural evidence supports the prioritization of Ftsl G306V and AcrR E91K as candidate determinants, the possibility that other genomic changes contribute to the observed phenotype remains open. Further experimental studies, including the construction of isogenic mutants and functional characterization of individual and combined variants, will be required to clarify their relative contributions and potential epistatic effects.

Among the chromosomal variants identified, the *ftsI*:917G > T substitution emerged as the most coherent candidate. FtsI encodes PBP3, the primary target of many β-lactam antibiotics, including cefiderocol. Residue G306 is located immediately adjacent to the catalytic serine S307 responsible for nucleophilic attack on the β-lactam carbonyl during acylation. From a structural perspective, the replacement of glycine, whose R group is limited to a hydrogen atom, with valine introduces an isopropyl group in the side chain containing three additional carbon atoms (Cβ, Cγ1, and Cγ2). The newly introduced side-chain bulk reduces the available space in the immediate catalytic microenvironment (Fröhlich et al. 2022).

In the WT model, the distance between residue 306 and the hydroxyl oxygen of S307 allows an open and flexible catalytic pocket compatible with optimal cefiderocol positioning. In contrast, in the G306V MUT, certain rotamer conformations of the valine side chain reduce this functional separation by 2.51 Å, narrowing the active-site cleft (Addlagatta et al. 2006).

Angular geometry further supports impaired catalytic alignment. In the wild-type model, the optimal Oγ(S307)–C(carbonyl)–N(β-lactam) angle was predicted to be 109.58º, a configuration compatible with favourable nucleophilic attack geometry. In the G306V mutant, however, this distribution shifted towards a wider value, indicating suboptimal alignment of the β-lactam carbonyl relative to the nucleophile. In enzymatic systems where acylation kinetics are highly sensitive to geometric precision, deviations of 10–20° (Robertson et al. 2022), as observed here, can translate into substantial reductions in acylation efficiency (k₂/K), even in the absence of major conformational disruption.

Moreover, docking simulations revealed significant differences between the WT and MUT models. In the WT structure, the average distance between the carbonyl carbon of the β-lactam ring and the Oγ atom of S307 in the conformation preceding acylation was 3.08 to 3.15 Å. This distance is well within the range of effective acyl-enzyme formation. In the MUT model, this distance increased to an average of 4.73 and 4.48 Å, frequently exceeding the optimal threshold for efficient nucleophilic attack in the majority of the 30 poses evaluated, suggesting a disrupted docking environment between the molecules. The proportion of docking poses that fulfilled both criteria (i.e., ≤ 3.5 Å) decreased substantially (93% in the WT model versus 10% in the MUT).

Importantly, cefiderocol possesses a bulky side chain, including a catechol moiety essential for siderophore-mediated uptake (Smith et al. 2024). Proper positioning of this extended structure within the PBP3 active-site requires adequate steric accommodation. The additional volumetric constraint introduced by valine at position 306 may cause subtle reorientation of the antibiotic within the catalytic pocket, increasing the average nucleophile–electrophile separation and destabilizing the conformation preceding acylation. This model is consistent with the resistant isolate’s preserved viability in vivo and in vitro. The global architecture of PBP3 remains intact, and essential transpeptidase function is likely to be maintained. The effect of G306V therefore appears to be a finely tuned modulation of catalytic nanogeometry that selectively impairs cefiderocol engagement without a catastrophic loss of enzymatic activity. Such subtle target adaptation represents a plausible evolutionary strategy under prolonged antibiotic exposure, particularly when alternative mechanisms (i.e., iron-uptake disruption) are either unavailable or associated with greater fitness costs. However, the precise nature of this effect remains speculative and should be interpreted with caution. Although our structural modelling and docking analyses support the plausibility of altered antibiotic-target interactions, dedicated in vitro structural and biophysical studies will be required to determine the direction and magnitude of this substitution on the interaction between PBP3 and cefiderocol. (Klein et al. 2022; Zhang et al. 2022; Simner et al. 2022).

Although β-lactamase-mediated modulation of cefiderocol susceptibility has been documented (Amadesi et al. 2024b; Freiberg et al. 2024), particularly involving SHV extended-spectrum β-lactamases, KPC variants, and certain NDM alleles, our data do not support the emergence of new carbapenemase variants or ESBL amplification that could explain the resistant phenotype. Nevertheless, the cefiderocol MIC of isolate 1268564, measured at 1 mg/L, was slightly above the epidemiological cutoff value reported for other Enterobacterales in the EUCAST database, highlighting the role of metallo-β-lactamases in cefiderocol inactivation (Buzgó et al. 2025).

Similarly, porin loss (e.g., OmpK35 inactivation), which has also been reported in the literature as being linked to reduced susceptibility (Tristancho-Baró et al. 2025b), was not observed in our paired comparison. The absence of canonical iron-transport alterations further distinguishes this case from the predominant mechanism described in global molecular epidemiology studies.

The presence of the FtsI V192G substitution in isolates SAMN34122257 and SAMN04014907, which are phylogenetically related yet genetically divergent and with clearly distinct resistomes, may suggest the possibility of clonal expansion of strains harbouring *ftsI* variants (Coppi et al. 2022; Schaufler et al. 2023). Furthermore, the six-fold higher cefiderocol MIC observed in SAMN34122257 with respect to SAMN04014907, reinforces the concept of the multifactorial nature of cefiderocol resistance and highlights the importance of target-site alterations as a potential secondary adaptive event in the development of resistance. However, in a broader context, the FtsI:G306V substitution appears to be infrequent in publicly available cefiderocol-resistant genomes, suggesting that, while target-mediated resistance may not represent a dominant population-level pathway, it can emerge under sustained selective pressure in individual hosts (Smith et al. 2024). Surveillance studies across Europe have demonstrated substantial heterogeneity in cefiderocol resistance among high-risk clones such as ST147, ST307, and ST512, which often involve diverse combinations of permeability, enzymatic, and regulatory factors (Nordmann et al. 2022). Our findings align with this broader epidemiological landscape in which cefiderocol resistance is heterogeneous and flexible.

In the same line, the absence of *ftsI*:917G > T and *acrR*:271G > A in more than 2800 complete genomes deposited in the NCBI database suggests that changes in highly conserved proteins are rare in the global population. Nevertheless, the identification of these mutations in the study isolates, as well as in three isolates within the global resistant subset, suggests that they may arise under selective pressure and become fixed in the genome without severely compromising bacterial fitness (Kriz et al. 2024).

Another observation was the restoration of carbapenem susceptibility in the resistant isolate. The recovery of carbapenem susceptibility following the emergence of cefiderocol resistance has been reported previously and is most commonly attributed to the altered hydrolytic profiles of specific KPC variants against different β-lactams (Hanna et al. 2025). Consistent with this observation, Castillo-Polo et al. reported that KPC-31 conferred resistance to ceftazidime–avibactam (MIC > 16/4 mg/L) while also increasing cefiderocol MICs by two- and three-fold dilution steps compared with KPC-3 and KPC-49, respectively (Castillo-Polo et al. 2024). Similar complex phenotypic patterns have been described during ceftazidime–avibactam therapy, highlighting the interconnectedness of resistance evolution and broader β-lactam susceptibility profiles (Amadesi et al. 2024a; Blanco-Martín et al. 2024; Amadesi et al. 2025). However, this explanation appears less likely in our case, as all β-lactamase genes, including the metallo-β-lactamase VIM, were genetically identical in both isolates. An alternative explanation could involve transcriptional downregulation of carbapenemase expression in the resistant isolate, although transcriptomic or gene-expression studies would be required to evaluate this possibility (Hanna et al. 2025).

Interestingly, Luu et al. recently demonstrated that the emergence of cefiderocol resistance and the concomitant restoration of carbapenem susceptibility could result from a combination of KPC evolution and reduced expression of PBP2 and PBP3 (Luu et al. 2025). The authors proposed that these changes reflected adaptive cell-wall remodelling in response to cefiderocol exposure, whereby reduced transpeptidase activity increased susceptibility to carbapenems by decreasing the availability of functional target enzymes within the periplasm. This hypothesis may fit our findings more closely, as it links alterations involving PBP3 to the phenotypic shifts observed across the β-lactams in the absence of detectable genetic changes in carbapenemase genes. Consequently, our results raise the possibility that modifications affecting PBP3 structure or function could contribute not only to cefiderocol resistance but also to collateral changes in β-lactam susceptibility, a hypothesis that warrants further investigation through transcriptomic and functional studies. Although speculative, altered PBP3 geometry could modify competitive binding dynamics among β-lactams, potentially influencing relative inhibitory efficiencies and generating collateral susceptibility shifts. The shift in tobramycin susceptibility might be related to the high substitution rate observed in 1303396 *aadA1*.

Paired analysis of susceptible and resistant isolates recovered from the same patient provides a powerful framework to identify adaptive mutations while minimizing background genomic variability (Klein et al. 2022; Tascini et al. 2024; Amadesi et al. 2024a). Combined with the genomic profiling and mechanistic analysis performed, this constitutes the main strength of this work, albeit important caveats: the conclusions are based on comparative genomics and in silico structural modelling rather than direct biochemical validation through allelic exchange or kinetic acylation assays. Furthermore, transcriptomic and crystallographic analyses were not performed, but are highly encouraged for future research aiming to clarify the role of target modification in cefiderocol resistance. Nonetheless, the paired within-host framework provides a robust temporal association between selective pressure and genetic adaptation.

## Conclusion

In summary, our data suggest that the emergence of cefiderocol resistance in vivo might be mediated through a target-mediated mechanism involving a substitution in the catalytic site of PBP3. Although disruption to the siderophore pathways remains the dominant mechanism described in the literature, this case presents a grounded hypothesis in which a subtle structural adaptation of the drug target can significantly impair the interaction between cefiderocol and PBP-3. However, it is important to emphasize that these findings are derived from comparative genomic analyses and in silico mechanistic modelling. As such, they provide a biologically plausible hypothesis regarding the genomic basis underlying the resistant phenotype observed in vitro, but they do not allow definitive conclusions regarding causality. Establishing a direct causal relationship would require additional experimental validation, including the construction and characterization of isogenic mutants as well as in vitro structural studies comparing the interactions of wild-type and mutant PBP3 proteins with cefiderocol. Such studies could include high-resolution structural approaches, quantitative binding assays, or kinetic analyses to determine whether the predicted geometric alterations effectively translate into reduced target affinity, impaired acylation efficiency, or other changes affecting antibiotic activity. The findings presented here may therefore serve as a foundation for future studies aimed at testing and refining this hypothesis. Incorporating analysis of *ftsI* variants within or proximal to the active-site into genomic surveillance frameworks may improve resistance detection for this last-resort antibiotic.

## Supplementary Information

Below is the link to the electronic supplementary material

ESM 1 (XLSX 10.7 KB)

ESM 2 (XLSX 11.0 KB)

ESM 3 (XLSX 18.6 KB)

ESM 4 (XLSX 14.5 KB)

ESM 5 (XLSX 10.8 KB)

## Data Availability

Data were submitted to GenBank on 05 Mar 2026 under Submission ID SUB16043148. Sequences can be found under BioProject accession PRJNA1432715. BioSample accession are SAMN56347749 (1268564) and SAMN56347750 (1303396). Genomes accessions numbers are JBVYQC000000000 (1268564) and JBVYQB000000000 (1303396). This Whole Genome Shotgun project has been deposited at DDBJ/ENA/GenBank under the accessions JBVYQC000000000 and JBVYQB000000000. The version described in this paper are versions JBVYQC010000000 and JBVYQB010000000.
